# Kinesin light chain-1 serine-460 phosphorylation is altered in Alzheimer’s disease and regulates axonal transport and processing of the amyloid precursor protein

**DOI:** 10.1186/s40478-019-0857-5

**Published:** 2019-12-05

**Authors:** Gábor M. Mórotz, Elizabeth B. Glennon, Jenny Greig, Dawn H. W. Lau, Nishita Bhembre, Francesca Mattedi, Nadine Muschalik, Wendy Noble, Alessio Vagnoni, Christopher C. J. Miller

**Affiliations:** 1 Department of Basic and Clinical Neuroscience, Institute of Psychiatry, Psychology and Neuroscience, King’s College London, 125 Coldharbour Lane Camberwell, London, SE5 9RX UK; 2 Division of Cell Biology, MRC Laboratory of Molecular Biology, Cambridge, CB2 0QH UK

**Keywords:** Axonal transport, Alzheimer’s disease, Kinesin-1, Kinesin light chain, Amyloid precursor protein, Calsyntenin-1, *Drosophila melanogaster*

## Abstract

Damage to axonal transport is an early pathogenic event in Alzheimer’s disease. The amyloid precursor protein (APP) is a key axonal transport cargo since disruption to APP transport promotes amyloidogenic processing of APP. Moreover, altered APP processing itself disrupts axonal transport. The mechanisms that regulate axonal transport of APP are therefore directly relevant to Alzheimer’s disease pathogenesis. APP is transported anterogradely through axons on kinesin-1 motors and one route for this transport involves calsyntenin-1, a type-1 membrane spanning protein that acts as a direct ligand for kinesin-1 light chains (KLCs). Thus, loss of calsyntenin-1 disrupts APP axonal transport and promotes amyloidogenic processing of APP. Phosphorylation of KLC1 on serine-460 has been shown to reduce anterograde axonal transport of calsyntenin-1 by inhibiting the KLC1-calsyntenin-1 interaction. Here we demonstrate that in Alzheimer’s disease frontal cortex, KLC1 levels are reduced and the relative levels of KLC1 serine-460 phosphorylation are increased; these changes occur relatively early in the disease process. We also show that a KLC1 serine-460 phosphomimetic mutant inhibits axonal transport of APP in both mammalian neurons in culture and in *Drosophila* neurons in vivo. Finally, we demonstrate that expression of the KLC1 serine-460 phosphomimetic mutant promotes amyloidogenic processing of APP. Together, these results suggest that increased KLC1 serine-460 phosphorylation contributes to Alzheimer’s disease.

## Introduction

Intracellular transport of proteins, organelles and other cargoes is an essential requirement for vertebrate cell function. This is particularly so for neurons since most neuronal proteins are synthesised in cell bodies and then have to be transported to their final functional destinations in axons, dendrites and synapses [6, 10, 15, 19, 31, 36]. Notably, the distances over which cargoes have to be trafficked through axons which can be over a metre in length in humans, present unique challenges for neuronal transport systems. Indeed, damage to axonal transport is known to contribute to Alzheimer’s disease, Parkinson’s disease and motor neuron diseases [6, 10, 15, 19, 36].

Changes in metabolism of APP are believed to contribute to Alzheimer’s disease; mutations in the APP gene cause some dominant familial forms of Alzheimer’s disease and proteolytic processing of APP generates amyloid-β peptide (Aβ) which is deposited as a pathology in the brains of Alzheimer’s disease patients [59]. APP is a type-1 membrane spanning protein and Aβ production involves successive cleavage of APP by β-site APP cleaving enzyme-1 (BACE1) and γ-secretase which cleave at the N- and C-termini respectively of the Aβ sequence. APP represents a key axonal transport cargo in Alzheimer’s disease. This is because disruption to anterograde axonal transport of APP is an early feature of Alzheimer’s disease, such disruption promotes amyloidogenic processing of APP and altered processing of APP itself perturbs axonal transport [28, 49, 50, 56]. Damage to axonal transport of APP has therefore been proposed to induce a toxic cycle of events that eventually lead to neuronal cell death in Alzheimer’s disease [50].

APP is transported anterogradely through axons on kinesin-1 motors [23, 50]. Most functional kinesin-1 comprises a heterotetramer of two kinesin-1 motor proteins (kinesin heavy chains) and two kinesin-1 light chains (KLCs). The heavy chains contain ATPase activity and generate motile force whereas the light chains (KLC1 and KLC2) are involved in attachment of cargoes [39]. A number of routes for attachment of APP to kinesin-1 motors have been described but a particularly important one involves the KLC ligand calsyntenin-1 (also known as alcadein-α) [11, 13, 30, 49, 56]. Calsyntenin-1 is a type-1 membrane spanning protein that binds directly to KLCs via C-terminally located tryptophan-aspartate motifs that interact with the tetratricopeptide repeat domain of KLCs [4, 12, 26]. Axonal transport of APP involves its loading onto calsyntenin-1 containing vesicles in the Golgi [56]. Thus, loss of calsyntenin-1 inhibits anterograde axonal transport of APP and also promotes BACE1 cleavage of APP to increase Aβ production [30, 49, 56]. Interestingly, calsyntenin-1 levels are reduced in Alzheimer’s disease brains and this suggests that calsyntenin-1 loss contributes to damaged APP transport in Alzheimer’s disease [56].

The mechanisms that underpin loading and release of APP containing vesicles to and from kinesin-1 motors are not properly understood but clearly represent key regulatory routes for controlling APP axonal transport. Notably, phosphorylation of serine-460 in KLC1 has been shown to inhibit its interaction with calsyntenin-1 leading to a reduction in calsyntenin-1 axonal transport [55]. KLC1 serine-460 is highly evolutionarily conserved [18, 55]. Moreover, these effects appear specific for calsyntenin-1 since KLC1 serine-460 phosphorylation does not influence binding and transport of a number of other KLC1 ligands [55]. Thus, via its effect on calsyntenin-1 binding, phosphorylation of KLC1 serine-460 may also regulate axonal transport of APP. Here, we address the role of KLC1 serine-460 phosphorylation in APP axonal transport and processing, and in Alzheimer’s disease.

## Materials and methods

### Plasmids

pCI-neo control empty vector was from Promega. Human APP (isoform 695) in pCI-neo, enhanced green fluorescent protein (EGFP)-tagged human APP (isoform 695) and FLAG-tagged wild-type KLC1 (isoform A; KLC1wt), and KLC1 in which serine-460 was mutated to aspartic acid (KLC1S460D) or alanine (KLC1S460A) have all been described previously [9, 43, 55]. Human APP isoform 695 fused in frame at its C-terminus to the yeast transcription factor GAL4 (APP-GAL4) was as described [22]. pG5-Luc in which firefly luciferase is driven by a GAL4 dependent promoter and pRL-CMV which expresses control Renilla luciferase were from Promega.

### Antibodies

The following primary antibodies were used in this study:

Mouse anti-total extracellular-signal-regulated kinase 1/2 (ERK1/2) (L34F12 Cell Signaling 1/2000), rabbit anti-active ERK1/2 (phosphorylated on threonine-202 and tyrosine-204) (Cell Signaling 1/2000), mouse anti-FLAG (M2 Sigma 1/2000), rabbit anti-total KLC1 (sc-25735/H-75 Santa Cruz 1/2000), mouse anti-neuron specific enolase (NSE Dako 1/50,000), mouse anti-tubulin (DM1A Sigma 1/20,000). Rabbit antibody to KLC1 phosphorylated on serine-460 was generated by immunisation with keyhole limpet hemocyanin coupled to peptide CKVDS^phos^PTVTTTLKNL in which serine-460 (S^phos^) was phosphorylated (Proteintech). The antibody was affinity purified against the peptide prior to use. Rabbit anti-total KLC1 (sc-25735/H-75) has been utilised in numerous previous studies e.g. [37, 41].

### Cell culture and transfection

Human embryonic kidney-293 (HEK293) cells were grown in Dulbecco’s modified Eagle’s medium with 4.5 g/l glucose (GE Healthcare) supplemented with 10% (v/v) fetal bovine serum and 2 mM L-glutamine. Cells were transfected using TurboFect (Thermo Scientific) or with polyethylenimine MAX (Polysciences) according to the manufacturer’s instructions. Cells were analysed 24 h post-transfection. Okadaic acid was from Santa Cruz and applied at 50 nM for 4 h.

Rat cortical neurons were obtained from embryonic day 17 embryos, plated on poly-L-lysine coated 18 mm diameter glass coverslips (Marienfield GmbH & Co.KG) and cultured in Neurobasal medium containing B27 supplement (Invitrogen), 2 mM L-glutamine, 100 IU/ml penicillin and 100 μg/ml streptomycin. Neurons were transfected using Lipofectamine 2000 (Invitrogen) (2 μl/μg DNA in Opti-MEM) according to the manufacturer’s instructions and analysed 24 h post transfection on DIV6 or 7.

### Preparation of human brain samples for SDS-PAGE and immunoblotting

Post-mortem human frontal cortex samples from control and pathologically confirmed cases of Alzheimer’s disease were obtained from the Medical Research Council Neurodegenerative Diseases Brain Bank, King’s College London. All tissue collection and processing were carried out under the regulations and licensing of the Human Tissue Authority, and in accordance with the Human Tissue Act, 2004. Post-mortem studies from some control, clinically non-demented individuals revealed early Braak stage pathologies. Frozen human brain tissues were prepared as 20% homogenates in ice-cold radioimmunoprecipitation assay (RIPA) buffer (50 mM Tris-HCl, pH 7.4; 150 mM NaCl; 1 mM EDTA; 1% (v/v) Triton X-100; 0.5% (w/v) sodium deoxycholate; 0.1% (w/v) SDS) with protease and phosphatase inhibitor cocktails (Complete and PhosStop, Roche) using a Bio-Gen PRO200 rotor-stator homogeniser (Pro Scientific) for 20 s. Following homogenisation, each sample was sonicated three times for 3 s before being centrifuged at 13,000 x g for 20 min at 4 °C. Supernatants were collected and protein concentrations determined using a bicinchoninic acid protein concentration assay kit (Pierce) according to the manufacturer’s instructions. Protein concentrations were adjusted to the same concentration in each sample by adding RIPA and SDS-PAGE sample buffers. Samples were stored at − 80 °C.

### SDS-PAGE and immunoblotting

Cells were harvested for SDS-PAGE and immunoblotting by scraping into SDS-PAGE sample buffer containing 2% (w/v) SDS, 100 mM dithiothreitol, 10% (w/v) glycerol, 0.1% (w/v) bromophenol blue plus protease in 50 mM Tris-HCl pH 6.8 and heating to 96 °C for 5 min. Human post-mortem brain samples were heated to 96 °C for 10 min prior to SDS-PAGE. Samples were separated on 8 or 12% gels using Mini-PROTEAN 3 gel electrophoresis systems (Bio-Rad) with a discontinuous buffer system. Separated proteins were transferred to BioTrace NT nitrocellulose membrane (0.2 μm pore size; Pall Corporation) using a Mini Trans-Blot electrophoretic transfer cell (Bio-Rad) for 16 h. Membranes were blocked with Tris-HCl buffered saline (TBS) containing either 5% (w/v) milk powder or 5% (w/v) bovine serum albumin, or with Odyssey TBS blocking buffer (Li-Cor Biosciences) for 1 h. Membranes were probed with primary antibodies in blocking buffers supplemented with 0.1% (w/v) Tween-20 (TBS/Tween-20), washed in TBS/Tween-20 and incubated with horseradish peroxidase (HRP)-conjugated secondary antibodies in wash buffer, and developed using an enhanced chemiluminescence development reagent (GE Healthcare) and detected using a BioRad ChemiDoc MP Imaging system. Alternatively, blots were incubated with IRDye-conjugated secondary antibodies in wash buffer and proteins visualised using an Odyssey CLx near infrared imaging system (Li-Cor Biosciences). KLC1 signals obtained from human brain samples were normalised to NSE signals from the same membrane. Both the BioRad ChemiDoc MP and Odyssey CLx near infrared imaging systems provide signals within the linear range and only such values were used for quantification.

### *Drosophila melanogaster* studies

All *Drosophila* stocks were cultured on Iberian food as described [53]. The following *Drosophila* strains were obtained from the Bloomington Drosophila Stock Center (Indiana University, IN): *Appl-Gal4* (BL#32040); *UAS-APP::YFP* (BL#32039); *nos-phiC31int.NLS* (attP40, BL#25709); *w*^*1118*^ (BL#5905). *TM3/TM6b* and *nos-Cas9* (BL#54591) stocks were gifts from Simon Bullock (MRC-LMB Cambridge). *nos-Cas9* and *nos-phiC31int.NLS Drosophila* were sequenced across the target region of the *Klc* gene to ensure no polymorphisms were present compared to the reference genome sequence that might interfere with the production of mutant *Klc Drosophila*.

KLC1 serine-460 and surrounding sequences are highly evolutionarily conserved and in *Drosophila* the homologous residue is KLC serine-433 [18, 55]. *Drosophila* KLC serine-433 was altered to aspartate using type II clustered regularly interspaced short palindromic repeat (CRISPR)/CRISPR-associated (Cas) mutagenesis. The guide RNA (gRNA) protospacer sequence directing Cas9-mediated cleavage was introduced by annealing the following oligonucleotides 5′- GTCGTGGCATAAGGCCGCTAAAG-3′ (top strand) and 5′-AAACCTTTAGCGGCCTTATGCCA-3′ (bottom strand) into the BbsI site of plasmid pCDF3 [44]. Potential off-target hits were evaluated using CRISPR target finder (http://tools.flycrispr.molbio.wisc.edu/targetFinder/) and E-CRISP (www.e-crisp.org/E-CRISP/). The gRNA efficiency score was calculated with the CRISPR Efficiency Predictor (http://www.flyrnai.org/evaluateCrispr/). The *gRNA-Klc* construct was integrated into the attP40 (25C6) landing site by phiC31 integrase-mediated transgenesis following embryo injection.

The single stranded DNA oligonucleotide donor (ssODN) for homology-directed repair was designed to anneal to an asymmetric region − 91/+ 36 bp (i.e. proximal/distal) from the protospacer adjacent motif (PAM) site and complementary to the “target” strand (i.e. the strand targeted by the gRNA) [46]. The ssODN sequence was 5′-CATATGGCGAGTACGGCGGTTGGCATAAGGCCGCTAAAGTAGATGACCCCACGGTCACAACCACTCTAAAAAATCTGGGAGCACTTTACCGACGTCAAGGCATGTTTGAAGCGGCCGAAACCCTGGA-3′ (4 nM Ultramer® DNA, Integrated DNA Technologies). The PAM site was mutated to prevent further Cas9 cleavage after the introduction of the desired mutation without a change to the amino acid sequence of the product. The ssODN was delivered in *nos-cas9/+; U6–3-gRNA-Klc/+* embryos 0.5-1 h after egg laying as a 500 ng/μl solution in H_2_O as previously described [44].

To identify KLCS433D mutant *Drosophila*, 4 G0 flies derived from the ssODN injected embryos were crossed to the TM3/TM6b balancer stock and the progeny of these flies screened by direct sequencing of the *Klc* gene. Briefly, a 582 bp region of the *Klc* gene encompassing the mutant site was amplified by PCR and sequenced as described [44]. The primer sequences were 5′-AAGCAACTTAACAATCTCGCCCTGCTC-3′ (Forward) and 5′-CGCATTCTTCTCCTCAGAGAAATCCAAATCC-3′ (Reverse). All founder animals and 12 of 23 offspring (52%) transmitted the mutation. G2 animals bearing the desired mutation were then backcrossed for 10 generations to an isogenic *w*^*1118*^ strain to minimise the possibility of off-target effects due to non-specific binding of the gRNA. During backcrossing, direct DNA sequencing of a PCR generated region of *Klc* was again used to identify mutant *Drosophila*.

For imaging of APP-YFP in the homozygous KLCS433D background (termed *KlcS433D*), *Appl-Gal4*; *KlcS433D/TM3* virgin females were crossed with *UAS-APP::YFP/CyO*; *KlcS433D/TM3* males. The control genotype involving wild-type *Klc* was generated by crossing *Appl-Gal4* virgin females to *UAS-APP::YFP* males.

### Quantification of APP transport by time-lapse microscopy

Axonal transport of APP-EGFP in living rat cortical neurons was monitored essentially as described previously for analyses of APP-EGFP and other fluorescent protein-tagged cargoes [1, 40, 41, 55–57]. APP-EGFP was imaged using either a Zeiss Axiovert S100 microscope driven by MetaMorph (Molecular Dynamics) and a 40x Plan-Neofluar 1.3NA objective, and a Photometrics Cascade-II 512B36 electron-multiplying charge-coupled device camera or alternatively, a Nikon Eclipse Ti-E microscope driven by NIS-Elements AR software and equipped with Intenslight C-HGFI light source, CFI Apo Lambda S 60x/1.40NA objective and an Andor Neo scientific complementary metal-oxide-semiconductor camera (Andor Technology) [41, 56, 57]. Filter sets were from Chroma Technology. APP-EGFP was imaged 24–36 h post-transfection in Ibidi μ-dishes or by mounting coverslips in a Ludin imaging chamber (Life Imaging Services) filled with external solution (145 mM NaCl, 2 mM KCl, 5 mM NaHCO_3_,1 mM MgCl_2_, 2.5 mM CaCl_2_,10 mM glucose in 10 mM HEPES pH 7.0). Temperature was maintained at 37 °C during imaging using either a Box Microscope temperature control system (Life Imaging Systems) for the Zeiss microscope or a microscope incubation chamber (Solent Scientific) for the Nikon microscope. Movements were recorded at 1 s time-lapse intervals and 100 ms exposure times. Kymographs were created using the Straighten and Kymograph plugins of ImageJ (developed by Wayne Rasband, National Institute of Health, Bethesda USA). Overall velocities for each run were calculated using the KymoAnalyser ImageJ macro package [42]. In line with previous studies, we chose cells expressing low levels of transfected APP-EGFP (as judged by the fluorescent protein signal) for analyses so as to avoid any possible artefacts produced by high levels of expression [1, 40, 41, 55–57].

APP-YFP movement was monitored in vivo in adult *Drosophila* sensory wing neurons essentially as described for other fluorescent protein-tagged cargoes [53, 54, 58]. Images were captured using a Nikon spinning disk system with a CSU-X1 scanning head (Yokogawa) and a Nikon Eclipse Ti-E inverted microscope equipped with a 60xCFI Apo/1.4NA objective and a Du 897 iXon Ultra electron-multiplying charge-coupled device camera (Andor). APP-YFP movement was monitored for 2 min with an acquisition rate of 1 frame/s. A temperature of 25 °C was maintained throughout imaging with a temperature unit and microscope enclosure (Okolab). Kymographs of APP-YFP were generated in Fiji/ImageJ with the KymoClear toolset from a 70 μm axonal region [32]. APP-YFP run numbers (defined as unidirectional movement in either anterograde or retrograde direction) and velocities were automatically analysed from the kymographs using the KymoDirect tool [32, 58]. Accuracy of the tracking was confirmed by manual inspection. Equal numbers of male and females were analysed and the data were derived from at least two independent *Drosophila* crosses.

### Luciferase reporter assays to monitor amyloidogenic APP processing

Amyloidogenic processing of APP was monitored in HEK293 cells essentially as described using an APP-GAL4 reporter assay that drives GAL4 upstream activator sequence (GAL4-UAS)-dependent expression of firefly luciferase [22]. Briefly, APP fused in frame at its C-terminus to GAL4 was co-transfected into HEK293 cells with a GAL4-UAS-firely luciferase reporter gene (pFR-Luc), Renilla transfection efficiency control plasmid (pRL-CMV) and either KLC1wt or KLC1S460D. BACE1, α-secretase and γ-secretase cleavage of APP releases the APP intracellular domain fused to GAL4 which translocates to the nucleus to regulate expression of firefly luciferase. Transfection efficiency normalised firefly luciferase signals thus provide a readout for amyloidogenic processing of APP. Cells were transfected in 24 well plates at 70–80% confluence with 0.25 μg each of APP-GAL4, pFR-Luc and either KLC1wt or KLC1S460D, along with 0.025 μg of pRL-CMV Renilla control plasmid. Cells were transfected as described above (see Cell culture and transfection). Luciferase signals were developed 24 h later using a Dual-Glo Luciferase assay kit (Promega) according to the manufacturer’s instructions and quantified using a Promega GloMax Navigator luminometer. Full details of the assay including design and construction of plasmids have been described previously [22].

### Aβ assays

Aβ (1–38), Aβ (1–40) and Aβ (1–42) levels were measured in conditioned media from HEK293 cells co-transfected with APP+KLC1wt or APP+KLC1S460D using a V-PLEX Plus Aβ peptide panel 1 (6E10) kit (Meso Scale Discovery) according to the manufacturer’s instructions. Cells were co-transfected in 24 well plates at 70–80% confluence with 0.25 μg of each plasmid as described above (see Cell culture and transfection). Conditioned media was harvested 24 h post-transfection and centrifuged at 1200 x g for 5 min at 4 °C. Supernatants were transferred into an ELISA plate and incubated for 1 h to capture Aβ species. Plates were then washed three times with PBS supplemented with 0.05% (v/v) Tween-20 and incubated with detection antibody for 2 h. Following three washes, read buffer was added to the samples and plates were analysed using a Meso Sector S 600 plate reader (Meso Scale Discovery).

### Statistical analyses

Statistical analysis was performed using Excel (Microsoft Corporation) and Prism software (version 7.02; GraphPad Software Inc.). Statistical significance was determined by t-tests or analyses of variance (ANOVA) followed by post-hoc test as described in the figure legends.

## Results

### KLC1 levels are reduced and the relative levels of KLC1 serine-460 phosphorylation are increased in post-mortem Alzheimer’s disease frontal cortex

**Fig. 1:**
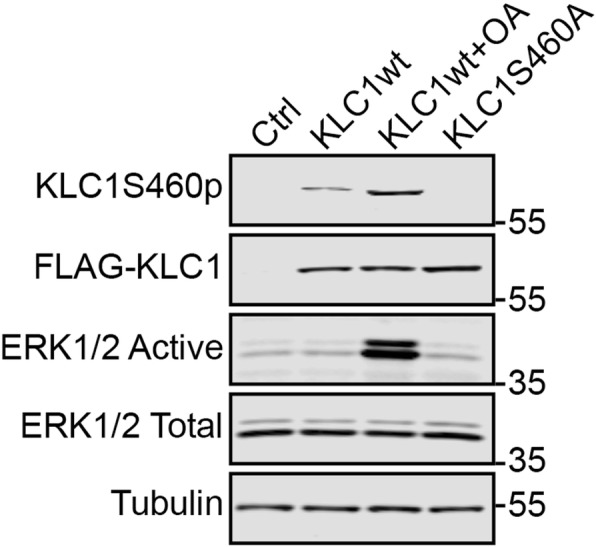
Characterisation of KLC1 serine-460 phospho-specific antibody. HEK293 cells were transfected with either control vector, FLAG-KLC1S460A or FLAG-KLC1wt and treated with either vehicle or okadaic acid (OA) as indicated. The different samples were then probed on immunoblots with antibodies to serine-460 phosphorylated KLC1 (KLC1-ser460p), FLAG to detect total KLC1 (FLAG-KLC1), active and total ERK1/2, and tubulin. The KLC1 serine-460 phospho-specific antibody detects KLC1wt but not KLC1S460A and signals with the antibody are increased in KLC1wt cells treated with okadaic acid that activates the KLC1 serine-460 kinase ERK1/2

To facilitate studies of KLC1 serine-460 phosphorylation in human Alzheimer’s post-mortem tissues, we generated a rabbit polyclonal antibody that recognises phosphorylation of KLC1 on this residue by immunisation with a KLC1 peptide in which serine-460 was phosphorylated. To demonstrate the specificity of this antibody, we transfected HEK293 cells with control vector, FLAG-KLC1wt or FLAG-KLC1S460A in which serine-460 was mutated to alanine to preclude phosphorylation. We also treated FLAG-KLC1wt transfected cells with the serine/threonine phosphatase inhibitor okadaic acid which activates ERK so as to increase KLC1 serine-460 phosphorylation; KLC1 serine-460 is targeted by ERK [55]. Probing of these samples on immunoblots with the KLC1 serine-460 phospho-specific antibody revealed that okadaic acid treatment increased FLAG-KLC1 signals but that signals were abolished in FLAG-KLC1S460A transfected cells (Fig. 1). These data are consistent with the antibody specifically recognising KLC1 phosphorylated on serine-460.

**Fig. 2:**
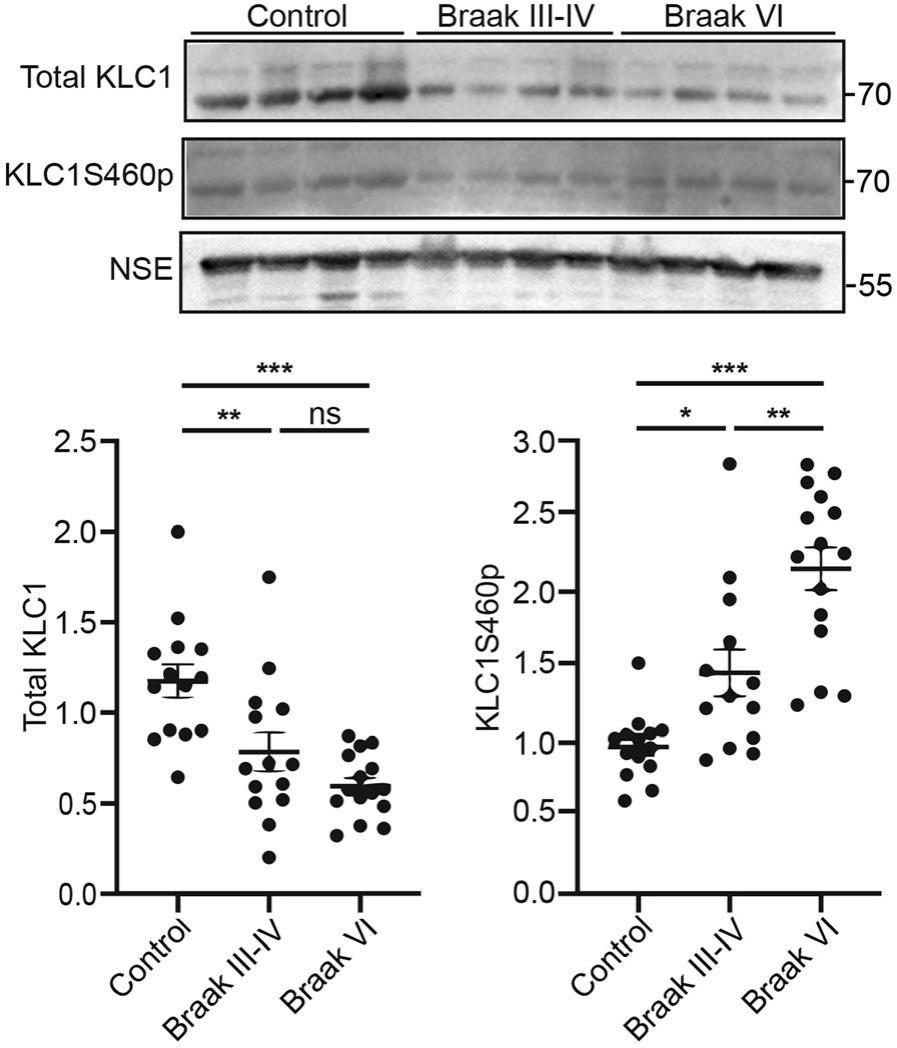
KLC1 levels are reduced and the relative levels of KLC1 serine-460 phosphorylation are increased in Alzheimer’s disease frontal cortex. Representative immunoblots showing total KLC1, KLC1 serine-460 phosphorylation (KLC1S460p) and NSE levels in post-mortem human control (Ctrl) and Alzheimer’s disease frontal cortex. Braak stages are indicated. Graphs show quantification of total KLC1 and KLC1 phosphorylated on serine-460 in the different samples. KLC1 and KLC1S460p signals were normalised to NSE levels. These normalised KLC1 levels were then used to quantify changes to total KLC1 and KLC1S460p (expressed as the ratio of KLC1S460p/total KLC1). Data were analysed by Welch’s ANOVA and Games-Howell post hoc test. *N* = 13–15, error bars are s.e.m., *p < 0.05 **p < 0.01, ***p < 0.001, ns not significant

We next utilised this antibody to enquire whether KLC1 serine-460 phosphorylation might be altered in Alzheimer’s disease. To do so, we monitored both total and serine-460 phosphorylated KLC1 levels in post-mortem control and Alzheimer’s disease frontal cortex tissues by immunoblotting. Details of these human samples are shown in Table 1; there were no significant differences in age or post-mortem delay between the Alzheimer’s disease and control cases. We studied total KLC1 and serine-460 phosphorylated KLC1 levels in frontal cortex in control, Braak stage III-IV (mid dementia) and Braak stage VI (severe dementia) cases. We normalised total and phosphorylated KLC1 levels to the levels of NSE as described by others [27, 41, 52]. Compared to controls, total KLC1 levels were significantly reduced in frontal cortex in both Braak stage III-IV and Braak stage VI cases (Fig. 2). Moreover, following normalisation to total KLC1 levels, the levels of KLC1 phosphorylated on serine-460 were increased in both Braak stage III-IV and Braak stage VI cases (Fig. 2). These data suggest that both loss of KLC1 and increased KLC1 serine-460 phosphorylation may contribute to damaged APP axonal transport in Alzheimer’s disease.

**Table 1:** Data for human post-mortem samples showing age at death, sex, post-mortem delay and pathological diagnosis

Case Group	Autopsy Code	Sex	Age	Post-Mortem Delay (h)	Braak stage
Control	A002/13	M	90	45	–
Control	A007/15	F	74	64	II
Control	A033/11	M	82	47	I
Control	A046/12	F	92	9	II
Control	A063/10	F	90	50	II
Control	A105/14	F	77	21	–
Control	A158/14	F	73	27	I
Control	A209/13	M	80	55	II-III
Control	A213/12	M	78	24	III
Control	A308/09	M	66	52	–
Control	A319/14	F	90	44	II
Control	A346/10	F	84	34	I-II
Control	A359/08	F	80	3	I
Control	A407/13	F	80	22	II
AD Braak III-IV	A037/04	F	96	39	IV
AD Braak III-IV	A041/04	F	97	67.5	III-IV
AD Braak III-IV	A065/16	M	91	48	IV
AD Braak III-IV	A067/09	F	92	19.5	III
AD Braak III-IV	A078/13	M	86	52.5	IV
AD Braak III-IV	A097/13	M	82	28	IV
AD Braak III-IV	A101/08	F	92	29.5	IV
AD Braak III-IV	A189/07	F	83	41.5	IV
AD Braak III-IV	A223/12	F	83	22	IV
AD Braak III-IV	A232/16	F	95	47	IV
AD Braak III-IV	A282/11	M	93	13.5	IV
AD Braak III-IV	A374/14	M	88	79	III-IV
AD Braak III-IV	A378/14	M	98	53	IV
AD Braak III-IV	A381/16	M	84	86	IV
AD Braak VI	A008/12	M	66	41	VI
AD Braak VI	A064/16	F	93	49	VI
AD Braak VI	A087/16	F	89	38.5	VI
AD Braak VI	A100/15	F	73	30	VI
AD Braak VI	A105/13	F	81	17.5	VI
AD Braak VI	A166/12	F	86	25	VI
AD Braak VI	A171/14	M	84	67	VI
AD Braak VI	A226/16	F	69	73	VI
AD Braak VI	A258/16	M	67	39.5	VI
AD Braak VI	A289/13	M	83	22	VI
AD Braak VI	A331/15	M	86	38	VI
AD Braak VI	A342/14	F	84	27	VI
AD Braak VI	A355/14	F	79	31	VI
AD Braak VI	A377/14	F	85	79	VI
AD Braak VI	A380/13	F	81	20	VI

AD, Alzheimer’s disease; F, female; M, male

### Mutation of KLC1 serine-460 to mimic permanent phosphorylation disrupts axonal transport of APP in cultured rat cortical neurons

Reduction of KLC1 levels has been shown to impair APP axonal transport in rodent and *Drosophila* neurons and to exacerbate Alzheimer’s disease phenotypes [16, 45, 50]. To investigate how phosphorylation of KLC1 serine-460 might contribute to Alzheimer’s disease, we quantified axonal transport of APP-EGFP using time-lapse microscopy in living rat cortical neurons that were co-transfected with either FLAG-KLC1wt or FLAG-KLC1S460D in which serine-460 was mutated to aspartic acid to mimic permanent phosphorylation. There are many examples whereby replacing serine residues with a negatively charged residue such as aspartic acid accurately mimics the effect of phosphorylation of the site (e.g. [1, 2, 14, 34]. Indeed, mutation of KLC1 serine-460 to aspartate has already been shown to accurately mimic phosphorylation of this site so as to reduce KLC1 binding to calsyntenin-1 [55]. Kymographs were generated from the time-lapse movies, and these were used to calculate the percentages of total, anterograde and retrograde moving APP-EGFP cargoes as previously described [41, 50, 56, 57].

**Fig. 3:**
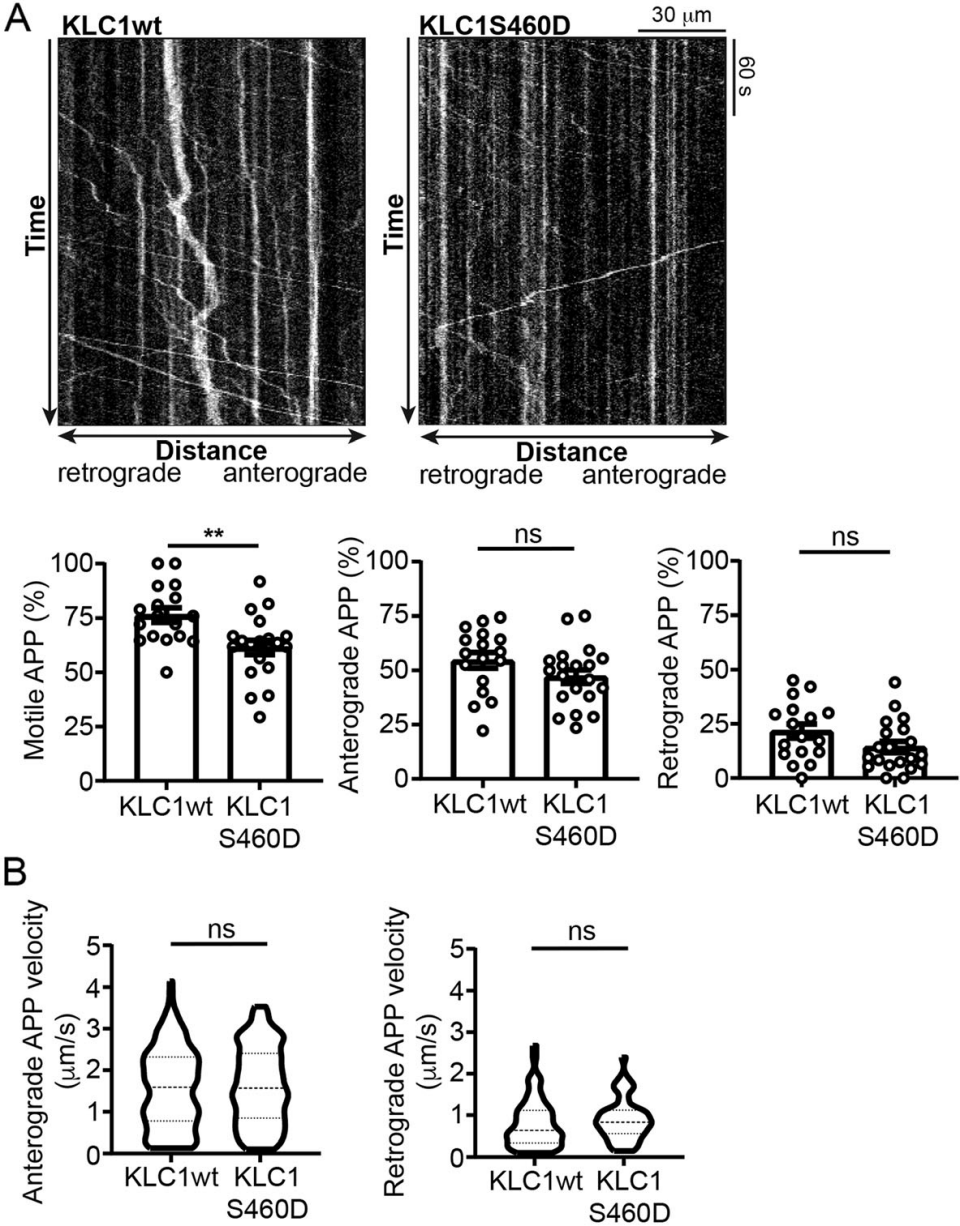
Mutation of KLC1 serine-460 to mimic permanent phosphorylation, inhibits axonal transport of APP in cultured rat cortical neurons. (**a**) Representative kymographs showing axonal transport of APP-EGFP in APP-EGFP+KLC1wt and APP-EGFP+KLC1S460D co-transfected neurons; scale bar and times are indicated. Bar charts show % for total, anterograde and retrograde APP-EGFP movement. *N* = 17 EGFP+KLC1wt and 20 APP-EGFP+KLC1S460D co-transfected neurons. Statistical significance was determined by Student’s t-test. Error bars are s.e.m.; **p < 0.01; ns not significant. (**b**) Violin plots show velocities of APP-EGFP movement in anterograde and retrograde directions in the different transfected cells. Median and interquartile ranges are indicated by hashed lines. *N* = 163 anterogradely and 62 retrogradely moving APP-EGFP in KLC1wt and 179 anterogradely and 54 retrogradely moving APP-EGFP in KLC1S460D co-transfected cells. Statistical significance was determined by Mann-Whitney U test; ns not significant

In APP-EGFP+FLAG-KLC1wt neurons, APP-EGFP movement was predominantly anterograde with mean speeds of 1.57+/− 0.95 μm/s in the anterograde direction and 0.81+/− 0.58 μm/s in the retrograde direction (mean+/−SD). These velocities and the bias towards anterograde movement are similar to those described previously for APP movement in rodent cortical neurons [51, 56]. However, compared to FLAG-KLC1wt co-transfected neurons, total (anterograde plus retrograde) APP-EGFP movement was significantly reduced in FLAG-KLC1S460D co-transfected neurons (Fig. 3a). Analyses of individual anterograde and retrograde transport revealed that although expression of FLAG-KLC1S460D appeared to reduce APP-EGFP transport in both directions, these reductions did not reach statistical significance (Fig. 3a). We also compared the anterograde and retrograde velocities of APP-EGFP movement in FLAG-KLC1wt and FLAG-KLC1S460D co-transfected neurons but detected no significant differences (Fig. 3b). Thus, compared to FLAG-KLC1wt co-transfected neurons, co-transfection of FLAG-KLC1S460D to mimic permanent phosphorylation inhibits total APP-EGFP axonal transport but does not alter the velocities of movement.

### Mutation of endogenous *Drosophila* KLC serine-433, the homologue of mammalian serine-460, to mimic permanent phosphorylation disrupts axonal transport of APP in vivo

**Fig. 4:**
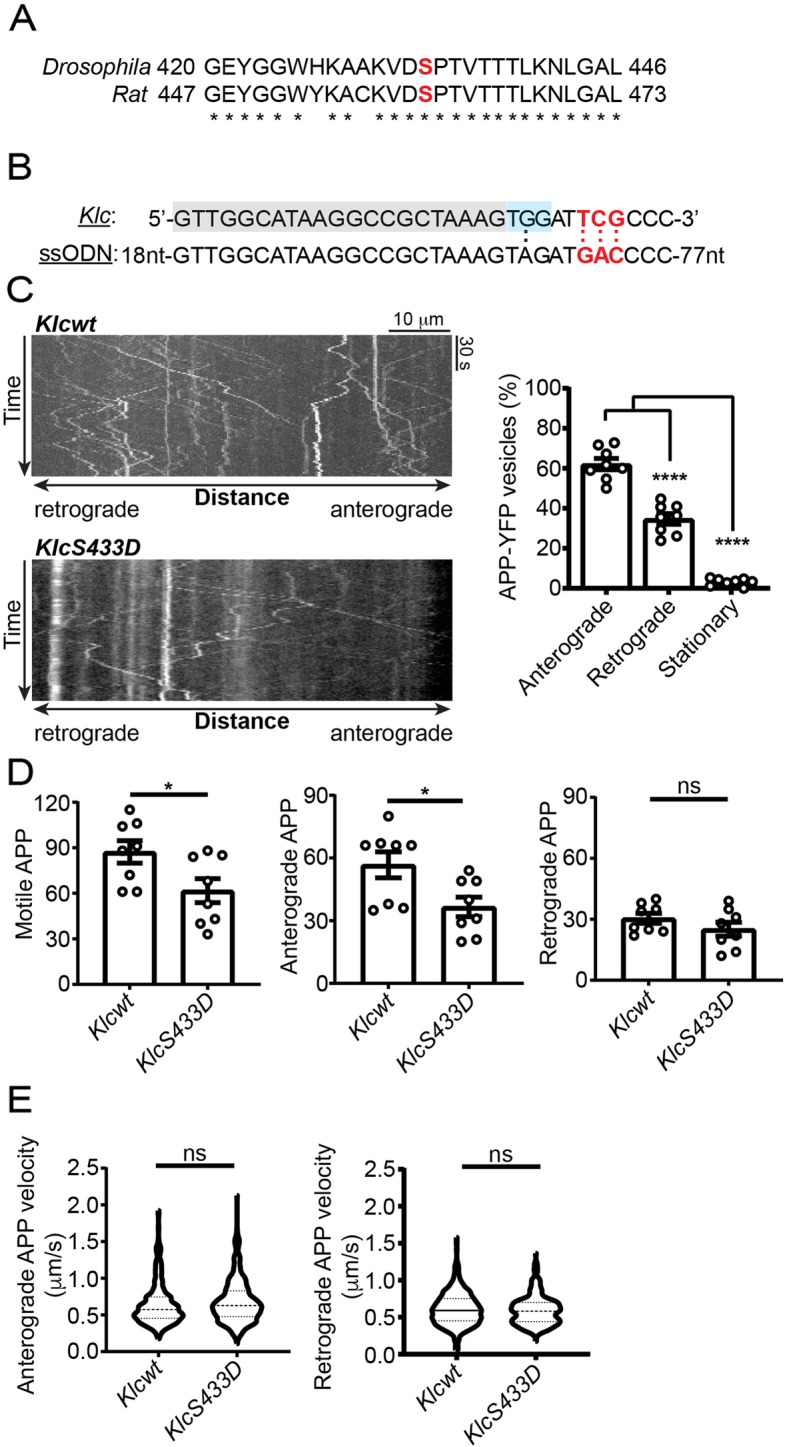
Mutation of endogenous *Drosophila* KLC serine-433 to mimic permanent phosphorylation inhibits axonal transport of APP in wing sensory neurons. (**a**) Alignment showing high conservation of the amino acid sequences encompassing rat KLC1 serine-460 and the homologous *Drosophila* KLC serine-433 (indicated in red). (**b**) CRISPR genome editing approach to mutate KLC serine-433 to aspartic acid. *Drosophila Klc* sequence (upper line) along with the ssODN (lower line) are shown. Serine-433 codon (KLC) and aspartic acid codon (ssODN) are shown in red, the protospacer sequence in grey shade and the PAM site in blue shade. A G-to-A transition is introduced to mutate the PAM. (**c**) Representative kymographs showing axonal transport of APP-YFP in *Klcwt* and *KlcS433D* homozygous backgrounds in 2-day old *Drosophila*; scale bar and times are indicated. Bar chart shows the relative proportions of stationary, anterograde and retrograde moving APP-YFP in the *Klcwt* background. (**d**) Bar charts show total, anterograde and retrograde APP-YFP movement runs in *Klcwt* and *KlcS433D* backgrounds. (**e**) Violin plots show velocities of APP-YFP runs in anterograde and retrograde directions in the different backgrounds. Median and interquartile ranges are indicated by hashed lines. *N* = 8 wings for each genotype. For velocity studies, *N* = 577 anterogradely and 347 retrogradely moving APP-YFP in *Klcwt*, and 343 anterogradely and 247 retrogradely moving APP-YFP in the *KlcS433D* background. Statistical significance was determined by one-way ANOVA with Holm-Sidak’s multiple comparison test in (**c**), Mann-Whitney U test in (**d**) and two-tailed Student’s t test in (**e**). Error bars are s.e.m.; **p < 0.01; **** *p* < 0.0001; ns not significant

*Drosophila* KLC displays a high degree of sequence and structural homology with mammalian KLC1 [18] and KLC1 serine-460 and flanking sequences are highly conserved between mammals and *Drosophila* (Fig. 4a). To complement the above studies in rat cortical neurons, we therefore analysed how mutation of the *Drosophila* homologue of mammalian KLC1 serine-460 (KLC serine-433) to mimic permanent phosphorylation (KLCS433D) affects APP axonal transport in vivo. *Drosophila* KLC serine-433 was mutated to aspartic acid by CRISPR/Cas9-mediated homology-directed repair (Fig. 4b). We then compared APP-YFP transport in transgenic *Drosophila* in which endogenous KLC was either wild-type or homozygous for KLCS433D. APP-YFP axonal transport was monitored in vivo in adult living sensory wing neurons essentially as described for other cargoes [53, 54].

In wild-type *Drosophila*, APP-YFP movement was bidirectional with mean speeds of 0.64+/− 0.01 μm/s in the anterograde and 0.61+/− 0.01 μm/s in the retrograde direction (mean+/−s.e.m.) (Fig. 4c). These transport characteristics are in line with previous findings of APP-YFP movement in *Drosophila* larvae segmental nerves [20, 50]. Anterograde and retrograde APP-YFP velocities were unaffected by the *KlcS433D* mutation (Fig. 4d). However, compared to wild-type *Drosophila*, both total and anterograde APP-YFP transport were significantly reduced in the *KlcS433D* mutants (Fig. 4d). Retrograde APP-YFP transport also displayed some reduction in *KlcS433D* mutant *Drosophila* but this did not reach significance. Thus, mutation of mammalian KLC1 serine-460 and its *Drosophila* homologue KLC serine-433 to mimic permanent phosphorylation inhibit axonal transport of APP in both mammalian cultured neurons and in vivo in *Drosophila.*

### Expression of mutant KLC1 serine-460 to mimic permanent phosphorylation promotes amyloidogenic processing of APP

**Fig. 5:**
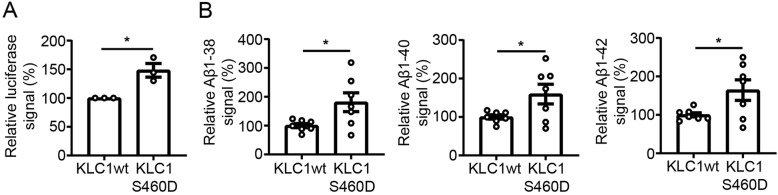
Expression of KLC1 serine-460 to mimic permanent phosphorylation promotes amyloidogenic processing of APP. (**a**) APP processing was monitored in HEK293 cells essentially as described using an APP-GAL4 reporter assay that drives GAL4-UAS-dependent expression of firefly luciferase [22]. Luciferase signals were normalised to co-transfected Renilla signals to correct for transfection efficiency. Bar chart shows relative luciferase signals in cells transfected with the GAL4-UAS-luciferase+Renilla reporters plus either APP-GAL4 + KLC1wt or APP-GAL4 + KLC1S460D as indicated. *N* = 15 from 3 independent experiments. Data were analysed by Student’s t test, error bars are s.e.m., *p < 0.05. (**b**) Expression of KLC1S460D increases Aβ production. Levels of Aβ (1–38), Aβ (1–40) and Aβ (1–42) we quantified in conditioned media from cells co-transfected with APP+KLC1wt or APP+ KLC1S460D. *N* = 7; data were analysed by Student’s t test, error bars are s.e.m., *p < 0.05

Disruption to kinesin-1 mediated APP transport has been shown to promote amyloidogenic processing of APP [28, 49, 50, 56]. We therefore monitored the effect of KLC1wt and KLC1S460D expression on APP processing using an APP-GAL4 dependent firefly luciferase reporter assay in which luciferase signals provide a readout for APP processing. γ-secretase processing of APP releases the APP intracellular domain fused to GAL4 which translocates to the nucleus to drive expression of a GAL4-UAS luciferase reporter [22]. Cells were co-transfected with APP-GAL4, firefly luciferase reporter and Renilla transfection efficiency control plasmids plus either KLC1wt or KLC1S460D. Compared to the KLC1wt co-transfected cells, transfection of KLC1S460D induced a significant increase in luciferase signals consistent with an increase in amyloidogenic processing of APP (Fig. 5a). To complement these assays, we also monitored Aβ (1–38), Aβ (1–40) and Aβ (1–42) levels in conditioned media of HEK293 cells co-transfected with APP+KLC1wt or APP+KLC1S460D. These assays revealed that expression of KLC1S460D significantly increased the levels of all these Aβ species (Fig. 5b).

## Discussion

Damage to axonal transport is an early pathogenic event in Alzheimer’s disease. APP represents a key axonal transport cargo since disruption to its transport promotes amyloidogenic processing of APP and altered APP processing itself damages axonal transport [28, 49, 50, 56]. Understanding how APP is transported through axons and how this transport is regulated are therefore important aspects of Alzheimer’s disease research.

APP is transported anterogradely through axons on kinesin-1 motors and a number of routes by which APP attaches to the motor have been described [7, 17, 24, 61]. One that has been linked directly to both APP transport and APP processing involves the KLC ligand calsyntenin-1 [11, 13, 30, 49, 56]. Here, APP is loaded onto calsyntenin-1 containing vesicles for transport by kinesin-1. Key regulatory events for this transport involve control of APP loading into the vesicle, and control of release of calsyntenin-1 (and hence vesicle associated APP) from KLCs.

Loading of APP onto calsyntenin-1 containing vesicles is believed to involve X11β, an adaptor coat protein located in the Golgi that interacts with both APP and calsyntenin-1 [3–5, 12, 21, 26, 35, 48, 56]. Thus, manipulating expression of X11β influences both APP axonal transport and its amyloidogenic processing [25, 29, 47]. The mechanisms regulating release of APP from kinesin-1 motors are less well characterised. However, phosphorylation of KLC1 serine-460 has been shown to inhibit binding and axonal transport of calsyntenin-1 [55]. Here, via the use of phospho-mimicking mutants, we provide evidence that KLC1 serine-460 phosphorylation also inhibits axonal transport of APP.

The KLC1S460D mutant we utilise in rat cortical neurons has already been shown to accurately mimic the effects of KLC1 serine-460 phosphorylation on binding and axonal transport of calsyntenin-1 [55]. We show that expression of mutant KLC1S460D also inhibits axonal transport of APP in these rat neurons. We also demonstrate that mutation of its *Drosophila* homologue KLC serine-433 to aspartate disrupts APP transport in vivo in *Drosophila* sensory neurons. In the cortical neurons, KLC1S460D reduced total (anterograde plus retrograde) transport but whilst both anterograde and retrograde transport appeared lower in the presence of KLC1S460D, these directional decreases did not reach significance. In *Drosophila*, KLCS433D reduced both total and anterograde axonal transport of APP. The more potent effect of KLCS433D on APP transport in *Drosophila* may be because the mutation is engineered into endogenous *Klc* whereas in the cortical neurons, the approach involved exogenous KLC1S460D so permitting additional kinesin-1 transport via endogenous non-phosphorylated KLC1.

Whilst we detected reductions in the proportions of moving APP in both the KLC1 phospho-mimicking rat cortical and transgenic *Drosophila* neurons, we did not observe any changes to the velocities of APP transport in either system. Such observations are consistent with known role of KLC1 serine-460 phosphorylation in the attachment of cargo (calsyntenin-1) as opposed to any modulation of kinesin-1 motor function [55].

We also present evidence that phosphorylation of KLC1 serine-460 affects amyloidogenic processing of APP. Using an APP-GAL4-dependent firefly luciferase reporter assay, we show that expression of KLC1S460D promotes γ-secretase processing of APP. We also show that KLC1S460D expression increases production of Aβ (1–38), Aβ (1–40) and Aβ (1–42) species. These findings are in line with the effects of KLC1S460D on APP transport since disruption to APP transport promotes amyloidogenic processing of APP [49, 50, 56]. Thus, phosphorylation of KLC1 serine-460 inhibits APP axonal transport and promote amyloidogenic APP processing.

Aside from ERK mediated phosphorylation of KLC1 serine-460, phosphorylation of other sites in KLCs have been shown to regulate axonal transport. Glycogen synthase kinase-3β (GSK3β) phosphorylation of a C-terminal region of KLC2 has been linked to reductions in transport of vesicles and other cargoes [33, 38]. GSK3β may also phosphorylate KLC1 although no sites have yet been identified [37]. However, other studies suggest that any effects of GSK3β on APP transport involve altering the activity of kinesin-1 motors rather than any changes to KLCs [60]. Phosphorylation of KLC1 threonine-466 has also been shown to influence transport of APP [8]. The kinase that targets KLC1 threonine-466 is not known but once identified, it will be interesting to investigate whether there are linked signaling pathways that regulate KLC1 threonine-466 and the nearby serine-460 site phosphorylated by ERK that we investigate here.

Finally, we show that KLC1 levels are reduced and the relative levels of KLC1 serine-460 phosphorylation are increased in Alzheimer’s disease frontal cortex. Reduced levels of both KLC1 and KLC2 have been described in late stage Alzheimer’s disease cortex (Braak stage V/VI) [37]. Our results thus complement these findings and show that loss of KLC1 occurs in earlier Braak stage cases; early pathogenic events are believed to be the most relevant to disease. There is also evidence that phosphorylation of KLC1 is increased in Alzheimer’s disease cortex although such studies have not identified altered phosphorylation of any specific residues [37]. Our findings are therefore the first to identify changes in phosphorylation of a defined site in kinesin-1 motor complexes in Alzheimer’s disease brain (KLC1 serine-460). Since we also present evidence that KLC1 serine-460 phosphorylation affects axonal transport and processing of APP, our results suggest that increased KLC1 serine-460 phosphorylation contributes to the pathogenesis of Alzheimer’s disease.

## Conclusions

Damage to axonal transport of APP is believed to contribute to the pathogenesis of Alzheimer’s disease. APP is transported anterogradely through axons on kinesin-1 motors and one route involves loading of APP into calsyntenin-1 containing vesicles; calsyntenin-1 is direct ligand for KLC1. The calsyntenin-1-KLC1 interaction is regulated by phosphorylation of KLC1 serine-460; phosphorylation promotes release of calsyntenin-1. Here we show that the expression of a phospho-mimicking mutant of KLC1S460 disrupts axonal transport of APP in rat cortical neurons and that a similar mutation disrupts transport of APP in vivo in *Drosophila* sensory wing neurons. We also demonstrate that the KLC1S460 mutant promotes amyloidogenic processing of APP. Finally, we show that KLC1 levels are reduced and the relative levels of KLC1 serine-460 phosphorylation are increased in Alzheimer’s disease frontal cortex, and that this occurs relatively early in the disease process. Our results suggest that increased KLC1 serine-460 phosphorylation contributes to Alzheimer’s disease.

## Data Availability

Experimental tools and data are available from the corresponding authors.

## Abbreviations

ANOVA: analysis of variance
APP: amyloid precursor protein
Aβ: amyloid β-peptide
BACE1: β-site APP cleaving enzyme-1
CRISPR: type II clustered regularly interspaced short palindromic repeat
CRISPR-associated: Cas
EDTA: Ethylenediaminetetraacetic acid
EGFP: enhanced green fluorescent protein
ERK: extracellular-signal-regulated kinase
GAL4-UAS: GAL4 upstream activator sequence
gRNA: guide RNA
GSK3β: Glycogen synthase kinase-3β
HEK293 cells: human embryonic kidney 293 cells
HRP: horseradish peroxidase
KLC: kinesin-1 light chains
KLC1S460A: kinesin light chain-1 serine-460 alanine mutant
KLC1S460D: kinesin light chain-1 serine-460 aspartic acid mutant
KLC1wt: wild-type kinesin light chain-1
KLCS433D: kinesin light chain serine-433 aspartic acid mutant
NSE: neuron specific enolase
OA: okadaic acid
PAM: protospacer adjacent motif
RIPA: radioimmunoprecipitation assay
s.e.m.: standard error of mean
SD: standard deviation
SDS-PAGE: sodium dodecyl sulphate polyacrylamide gel electrophoresis
ssODN: single stranded DNA oligonucleotide donor
TBS: Tris-HCl buffered saline
UAS: upstream activation sequence
YFP: yellow fluorescent protein
